# Microbiome and Diet Interplay: An Emerging Frontier in PDAC Diagnosis and Prevention

**DOI:** 10.3390/cancers18081292

**Published:** 2026-04-19

**Authors:** Birbal Singh, Francesco Marotta, Gorakh Mal, Rinku Sharma, Devi Gopinath, Gauri Jairath, Ajayta Rialch

**Affiliations:** 1 ICAR-Indian Veterinary Research Institute, Regional Station, Palampur 176061, Indiarinku.sharma@icar.org.in (R.S.); devi.gopinath@icar.org.in (D.G.); ajayta.rialch@icar.org.in (A.R.); 2 ReGenera R&D International for Aging Intervention, 20144 Milan, Italy

**Keywords:** PDAC, gut microbiome, dysbiosis, life style modifications, microbial therapies, co-biotics

## Abstract

Pancreatic ductal adenocarcinoma (PDAC), which remains undetected till advanced stages of progression, is becoming a more common and aggressive PC with high mortality rates. Trending unhealthy dietary habits, sedentary life, obesity, diabetes, high body mass index (BMI), and perturbed gut microbiota, also known as dysbiosis, increase the PDAC incidences. There is an urgent need to delve into reliable and cost-effective biomarkers and strategies to diagnose and prevent the disease. Microbial remedies and dietary phytonutrients that restore gut microbial balance may be the prospective preventive measures. This article intends to recommend the hypothesis that PDAC is incepted and characterized by microbial signatures and can be managed by integrating evidenced-based complimentary approaches entailing diet, nutraceuticals, microbial interventions and life style modifications with standard therapies. The futuristic longitudinal and mechanistic studies to unravel host–microbiota and the host–microbiome interactions will allow deeper aetiologic and mechanistic insights into PDAC to evolve therapies and recommendations for better pancreatic health.

## Simple Summary

Rather than being a distinct single disease, pancreatic cancer (PC) is a multifaceted disease that evolves gradually and dynamically in response to adverse local and environmental stresses emerging from pollutants, natural aging, involuntarily consumed xenobiotics, dybiosis of internal microbiota and prolonged metabolic diseases such as obesity and diabetes. The global cancer statistics indicate a globally rising burden of PC and the cancer-linked deaths. There is urgent need to diagnose pancreatic cancer at early stages through suitable microbiological and biochemical markers, and evolve preventive measures that are equitable and accessible, especially to low-income societies.

## 1. Introduction

In the present time of hectic and peripatetic life, humans are unavoidably exposed to multiple environmental hazards, biotic and abiotic stresses which affect health, and exert stress on physiology, immune system and functioning of different organs. The PC is a lethal malignancy, ranked as fourth leading cause of cancer-linked mortalities in developed countries. According to global cancer statistics for the year based on updated estimates from the International Agency for Research on Cancer (IARC), a Global Cancer Observatory, there were nearly 20 million new cases of cancers in 2022 including non-melanoma skin cancers (NMSCs), along with 9.7 million deaths [1]. The statistics imply that approximately one in five men develop cancer during their lifetime, whereas one in nine men and one in 12 women died from the cancer. In 2022 alone, the most frequently observed cancers were lung cancer (2.5 million or one in eight cancers worldwide) (12.4% of all cancers globally), followed by breast cancer (11.6%), colorectal cancers (CRC) (9.6%), prostate cancer (7.3%), and gastric cancer (4.9%). The global cancer incidences are likely to raise to 35 million by 2050 [1].

The number of people, including elderly persons, with cancers is rising in advanced and developed countries such as the United States of America. The age of longevity has increased as a result of timely detection of various diseases and healthcare management. As of 1 January 2025, about 18.6 million people in the USA had a history of cancer, and the figure is expected to rise to more than 22 million by 2035 [2].

The PDAC has poor prognosis due to the fact that it comprises complex dense fibrotic tumor microenvironment (TME) which hastens cancer progression and resists anticancer therapies, hence making it difficult to detect the disease at preclinical stages. Galectin-1 (Gal1), a glycan-binding protein abundantly expressed by activated pancreatic stellate cells (PSCs), is a key player of the PDAC progression. The nuclear Gal1 levels elevate in human PSCs in vivo as well as in cultured pancreatic cancer cell lines [3]. The Gal1 in fibroblasts regulates expression of cancer-associated *KRAS* gene, and sustains KRAS protein synthesis in fibroblasts, which, in turn, activates PSCs and leads to synthesis of pro-tumorigenic cytokines, endorsing tumor growth and consequent invasion to other tissues [3].

## 2. Pancreatic Cancer Epidemiology

PC is a rare but highly lethal disease affecting men and women in developed as well as poor nations. Depending on the type of cells involved, the pancreatic tumor may be exocrine, neuroendocrine or endocrine. Approximately 92% of pancreatic tumors are exocrine in origin, arising from the enzyme-producing acinar cells or ductal epithelial cells of the pancreas. The remaining 8% are pancreatic neuroendocrine tumors (PNETs), also known as islet cell tumors, which generally exhibit slower growth than exocrine pancreatic tumors.

People with healthy habits, i.e., being non-smokers, non-diabetic and/or non-alcoholic, may sometimes develop PC, implying the involvement of multiple factors in its commencement. According to the global, regional, and national burden, PC incidences from 1990 to 2021, and projections of risk factors to 2050, the PC incidences have increased from approximately 207,905 to 508,533, and the age-standardized incidence rate (ASIR) has increased from 5.47 to 5.96 per 100,000 human population [4]. Further, the global burden of PC, measured in terms of disability adjusted life years (DALY), has ascended from 5.21 million to 11.32 million [4]. In 2025, 2,041,910 new cancer incidences and 618,120 cancer-arbitrated deaths have been projected in the United States alone [5].

The global lifetime risk of developing PC is 0.89% (95% CI 0.88–0.89), ranging from 0.15% (0.130–0.18) in middle Africa to 2.06% (2.04–0.08) in Western Europe [1]. According to reports, among all cancers, PC ranks 12th in incidents, and 6th in cumulative death rates. The median survival is approximately 4 months with a 5-year survival of 13% [1,6]. 

## 3. What Causes PDAC?

Exact causes of PDAC are not yet fully understood. Advances in biochemical analysis techniques and computerized analysis of the data obtained has identified various factors that can be categorized as modifiable (e.g., tobacco smoking, high alcohol consumption, high BMI, diets including processed meat and saturated fats, chronic pancreatitis, *Helicobacter pylori*, and hepatitis C infection), and the non-modifiable factors (gender, ethnicity, ABO blood group, diabetes mellitus, familial history and genetic susceptibility to the disease) [7].

**Figure 1:**
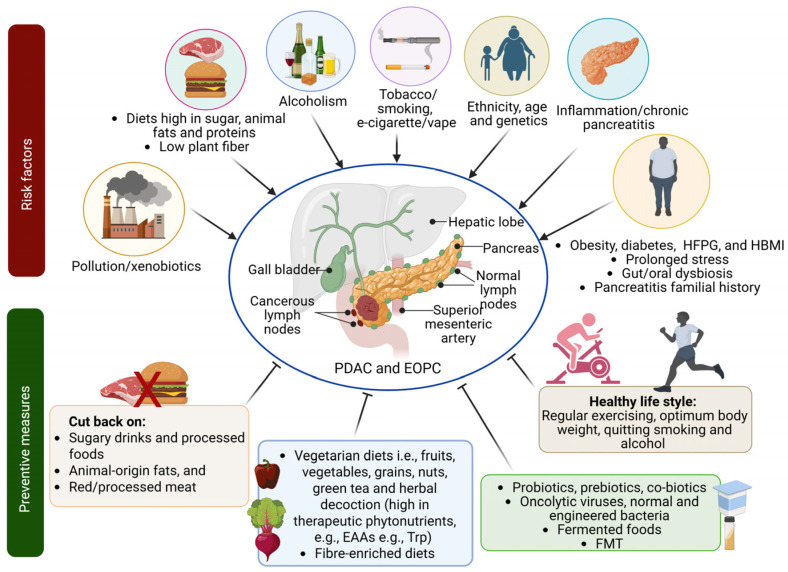
PDAC and EOPC risk factors and the salient preventive measures. There are several risk factors to which humans are exposed inadvertently. Active life style and physical exercise thwart inflammatory reactions in body. Findings from large-scale pooled analysis indicate that types of alcohol and ethnicity have a role. For instance, spirits and beer had increased risk, but wine had no significant risk. Compared to Asia, the participants from Europe, Australia and North America had higher risk of PDAC [19]. Abbreviations: EAAs—essential amino acids; EOPC—early onset pancreatic cancer; FMT—fecal microbiota transplantation; HBMI—high body mass index; HFPG—high fast plasma glucose; Trp—tryptophan. Created in BioRender. Singh, B. (2026) https://BioRender.com/dwk6q8r.

There is evidence of correlation between longstanding history of heavy alcohol consumption (≥80 g of alcohol/day for more than 5 years) and chronic pancreatitis, albeit the underlying mechanisms are yet to be elaborated [8]. Moreover, xenobiotics (e.g., mycotoxins, polycyclic aromatic hydrocarbons, cooking oil byproducts, dietary nitrosamines in processed foods, dietary volatile hydrocarbons, pesticides and heavy metals, etc.) induce deleterious cellular and metabolic alterations through oxidative stress and inflammation, leading to pancreatitis which in turn may serve as risk factor for PDAC over the time [9,10] (Figure 1).

Increasing consumption of Western diets high in animal fats and proteins, mostly the red meat, diabetes, obesity, metabolic syndrome sedentary life style, smoking, gum diseases, age, ethnicity, and gender (males being more susceptible) are some of the other noticeable factors responsible for PDAC [11,12]. Hereditary pancreatitis is also noted in some cases. Nearly 10% of PCs are considered as familial or hereditary [13]. High-risk familial history and genetic susceptibility include intraepithelial neoplasia, Peutz–Jeghers syndrome, hereditary pancreatitis, familial atypical multiple mole melanoma, familial PC, familial breast cancer, familial adenomatous polyposis and Lynch syndrome, etc. [14]. Deficiency of 25-hydroxyvitamin-D, sustained psychologic stress and smoking are among the other prominent risk factors. Lack of symptoms before PDAC at early stages, pro-tumoral and hyperfibrosis stroma developed by cancer-associated fibroblasts, high genetic and metabolic heterogeneity among patients, diagnosis at late stages, early lymphatic and blood spread, age of patient unfit for ablative surgery, poor blood supply, lack of actionable molecular targets, and typical immunosuppressive microenvironment of the tumor make it difficult to treat the PDAC [15,16,17].

A cohort study on role of dysglycaemia as a risk factor for PDAC, involving a total of 499,804 patients from the UK Biobank study, has shown that PDAC developed in a total of 1157 participants during 11.6 (10.9–12.3) years follow up. The dysglycaemia indicated by increased HbA1c levels was correlated with increased risk of PDAC, and the strength of the association between elevated HbA1c and incidence of PDAC was inversely proportional to the time from noticing dysglycaemia, and was significant for at least 60 months after HbA1c testing [18].

**Figure 2:**
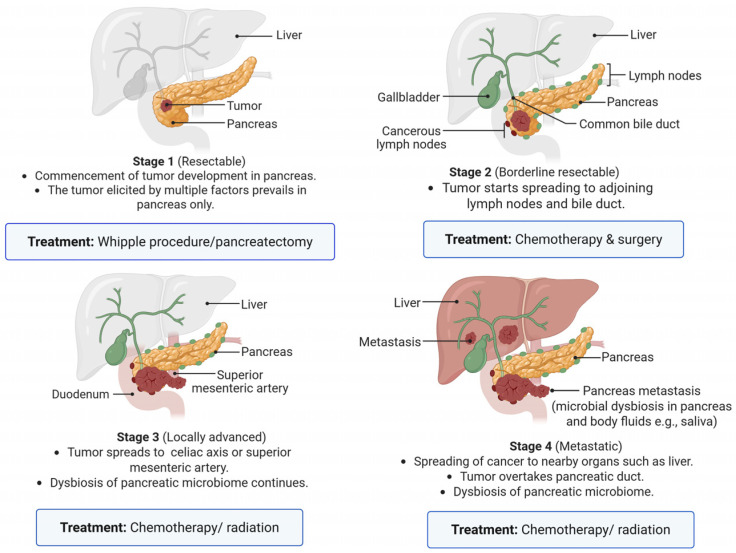
Progression and characteristic features of PDAC. Different stages are characterized by the tumor progression to adjoining organs. The PDAC has poor tumor perfusion leading to hypoxia which impedes drug delivery to the tumor and resists radiotherapy. The synthetic, gene-edited or engineered bacteria (probiotic) adapted to hypoxic or anaerobic environment may serve as prospective vehicles to deliver anti-tumor drug load [20]. Created in BioRender. Singh, B. (2026) https://BioRender.com/dwk6q8r.

The PDAC progresses in different stages (Figure 2). Initially, the tumor develops in the pancreatic duct which spreads to other organs such as the lymph nodes and liver at advanced stages. Preventive strategies for disease progression should be tailored according to the stage of the disease and the patient’s overall clinical condition.

## 4. Microbiome and PDAC Camaraderie

There has to be a balance between normal microbiota and the host health. Any perturbation in the normal microbiome causes moderate to severe health risks including behavior and cognitive health alterations. A large body of evidence indicates that an optimal gut microbiome eubiosis characterized by predominance of Bacteroidetes and Firmicutes, and paucity or low abundance of Proteobacteria, Actinobacteria and Verrucomicrobiota is essential for healthy digestive systems and gut health.

While gut barrier is essential to facilitate assimilation of nutrients into blood circulatory stream, and prevent translocation of pathobionts and their xenobiotic metabolites such as lipopolysaccharides (LPS), flagellin, colibactin and microbial mRNA from intestinal lumen to distant organs, disruption of gut barrier leads to inflammatory responses and related diseases. Colibactin-producing *Escherichia coli* (CoPEC) activates the immune system through tumor infiltrating lymphocytes (TILs), cytokines, immune cells and Toll-like receptors (TLRs), leading to activation of pro-inflammatory mediators activating STAT3 and NF-kB pathways, increasing cell proliferation and suppressing apoptosis, culminating in CRC [21].

With notable exceptions (e.g., CoPEC), the dysbiosis does not directly cause genotoxicity or mutagenic alterations, rather it activates immune system through pathways such as tumor infiltrating lymphocytes (TILs), TLRs, cytokines, and immune cells which collectively activate pro-inflammatory mediators activating STAT3 and NF-kB pathways, promoting cell proliferation and suppress apoptosis, ultimately culminating in carcinogenesis [21].

Certain cancer niches such as CRCs are readily colonized by CoPEC which induces genotoxicity (DNA double strand breaks, the severe form of DNA damage), mutations, genomic instability and cellular senescence. The infected cells synthesize a senescence-associated secretory phenotype (SASP) which is involved in tumor development in mouse models [22]. Although pancreatic microbiome shows quite low intrinsic biomass and thus being unlikely per se to trigger malignant transformation [22], it has been observed that the pancreatic juice had significantly depleted antibacterial activity in patients with chronic pancreatitis [23], and this may ease the entry of mutagenic intestinal metabolites into blood circulation. Indeed, this situation may be reconciled with a wider range of immunological reactions [24].

People with poor oral hygiene are more vulnerable to PC than the people with a healthier oral ecosystem and mouth. Experimental studies have corroborated the mouth-microbiome link where periodontal pathogen *Porphyromonas gingivalis* accelerated pancreatic intraepithelial neoplasia (PanIN) to PDAC in mice models followed by suggestive clinical data [25].

A significant deviation in microbial diversity was noted in the saliva of PC patients compared to healthy controls. The PDAC patients’ pancreas exhibited a distinct microbial profile, predominantly of Firmicutes, Proteobacteria, and Actinobacter after filtering the microbiome of the internal environment. The oral bacteria including *Anoxybacillus*, *Bacillus* and *Clostridium* were thought to have translocated from oral cavity to pancreatic tissue [26].

The microbial dybiosis is linked to various diseases such as inflammatory bowel syndrome, CRC and the diseases of various organs. 16S rRNA gene sequencing of patients with long-time survival (LTS) with active immune response had higher α-diversity (i.e., metrics describing the species richness, evenness or diversity within a sample) in the tumor microbiome, and characteristic intratumoral microbiome signature (*Pseudoxanthomonas-Streptomyces-Saccharopolyspora-Bacillus clausii*) [27]. The microbial diversity is high in LTS discovery as well as validation cohorts. Through human-into-mice fecal microbiota transplantation (FMT) experiments using microbial inoculums from short-term survivors (STS), LTS or control donors, it was possible to modulate the tumor microbiome and affect the tumor growth and tumor immune filtration, indicating that the PDAC microbiome composition with a cross-talk connection with gut microbiome can influence the host immune response and natural history of the disease [27]. The tumor microenvironment bacteria potentially modulate the tumor sensitivity to chemotherapy drugs such as 2′,2′-difluorodeoxucytidine, popularly known as gemcitabine. The drug is used to treat solid tumors including PC, non-small pulmonary cancer, breast cancer and bladder cancer. According to a study, of the 113 PDAC patients examined, 86 (76.1%) persons were positive for a specialized category of the intratumor bacterial species, mainly the Gammaproteobacteria that metabolize gemcitabine into an inactive form, the 2′,2′-difluorodeoxuuridine [28].

## 5. Biomarkers of PDAs

### 5.1. Gut and Intratumoral Microbiota as PDAC Signatures

The human microbiota has emerged as a key modulator of various health outcomes including inflammatory and non-inflammatory communicable diseases (NCDs). The microbial dysbiosis may contribute to pathophysiology of NCDs through metabolic and inflammatory pathways. The research into the human microbiome and its impact on health highlights how the commensal microbial perturbations elicit inflammation and promote metabolic disorders and cancers.

Historically considered as a sterile organ, the pancreas has its own distinct microbiome. The microorganisms originating from the oral cavity, stomach and intestine may translocate to and colonize the common bile duct and consequently migrate to the pancreatic duct [29]. The mechanisms of microbial translocation are speculated to be diverse, such as migration to the pancreatic ductal system via mesenteric venous drainage or lymphatic network [30]. Irrespective of their origin, depending on situations, the intratumoral microbiota may have a role in cancer prevention or progression.

Random Forest analysis showed elevated abundance of *Butyrivibrio*, *Agathobacter*, *Hafnia-Obseumbacterium*, Prevotellaceae NK3B31 group, *Methylobacterium*-*Methylobrum*, *Barnesiella* and *Ruminococcus gnavus* group, as markers of control samples, while CAG352 and *Lactobacillus* were the potential PDAC biomarkers [31]. Another study involving MiSeq sequencing analysis of gut microbial diversity of PC patients (85 nos.) and healthy controls (57 nos.) has reported abundance of certain pathogens (e.g., *Klebsiella*, *Selenomonas*, and *Veillonella*) and LPS-producing bacteria (e.g., *Enterobacter*, *Hallella*, and *Prevotella*), and reduced probiotics (*Butyrivibrio* spp.), butyrogenic bacterial phyla (*Anaerostipes*, *Blautia*, *Clostridium* IV, *Coprococcus*, and *Flavonifracter*) in PDAC patients [32].

Microbiome type and abundance unraveled using linear discriminant analysis effect size (LeFSe) and Random Forest analysis have been used as biomarkers of PC in humans. A 16S sequencing of fecal samples obtained from 22 patients with PDAC, 11 with intraductal papillary mucinous neoplasm (IPMN) and 24 controls have revealed abundance of Firmicutes and Proteobacteria in PDAC patients, while controls had an abundance of Bacteroidota [31]. LeFSe analysis revealed an abundance of *Escherichia-Shigella*, *Fusobacterium*, *Klebsiella*, *Sutterella*, *Eubacterium ventriosum* group, CAG 352, *Bifidobacterium*, *Odoribacter*, *Eubacterium ruminantium* group, *Ezakiella* and *Colidextribacter* in PDAC groups. On the contrary, *Bacteroides*, *Faecalibacterium*, *Agathobacter*, *Akkermansia*, *Subdoligranulum*, *Alistipes*, *Fusicatenibacter*, *Lachnospiraceae* UCG 004 and *Lachnospira* were more prevalent in the control group [31]. Although further studies toward this target are urgently proposed, the inferences available underscore the significance of in-depth understanding of the interplay between inflammation and microbiome in the context of PDAC.

More robustly, a cohort study on data from two epidemiological cohorts, i.e., the American Cancer Society Prevention Study-II Nutrition Cohort, and the Prostate, Lung, Colorectal, and Ovarian Cancer Screening Trial, involving 122,000 participants was carried out. Based on analysis of cohort patients whose oral samples were analyzed for microbial consortia, 445 patients developed PC over a median (IQR) follow-up of 8.8 (4.9–13.4) years. The whole genome shotgun sequencing and internal transcribed spacer sequencing followed by microbial risk scores (MRS) has shown that periodontal pathogens, namely *Porphyromonas gingivalis*, *Eubacterium nodatum*, and *Parvimonas micra*, and the fungi belonging to *Candida* genus were linked to increased PC risk [33].

16S rRNA amplicon analysis of saliva, duodenal fluid and pancreatic tissues of the stage I/II, III and IV PDAC patients has shown that salivary and duodenal microorganisms play a prominent role in shaping the PDAC microbial landscape. A thorough understanding of the role of these microorganisms and their role in PDAC pathogenesis will help to develop future therapies and preventive measures against PDAC [34]. Therefore, oral and tumoral microbiota can be useful as non-invasive biomarkers to identify the individuals at high risk of PDAC, potentially contributing to diagnosis and personalized healthcare.

### 5.2. Biochemical Markers

There is a need for minimally invasive biochemical and microbiological markers to diagnose PDAC during the early stages of development. Circulating cell-free RNA subtypes (e.g., DEGS1, KDELC1 and RPL23AP7) [34], tumor budding and E-Cadherin loss [35] and the carbohydrate antigen 19-9 (CA 19-9), a mucin-like glycoprotein discovered on the cancer cell surface, are the generally used biochemical markers of PDAC. The CA 19-9 duly approved by USFDA is a widely used blood biomarker to diagnose PDAC [36].

The use of CA 19-9 as potential biomarkers is impeded by its poor diagnostic specificity, yield of false-positives in benign biliary conditions, false-negatives (especially in Lewis-negative individuals), low early-stage sensitivity and incongruity to screen cancer in large human population. Therefore, there is a need to have alternative biomarkers to diagnose PDAC.

A multiplex proteomic analysis aimed to identify early detection of PDAC, based on proteomic analysis of blood proteins from stage I and II PDAC patients, and individuals at high-risk and the heathy controls, has outlined a subset of 41 proteins as PDAC biomarkers that significantly outperformed CA 19-9 in terms of AUC of 0.95, 84% sensitivity, and 95% specificity [37]. However, the above 41 biomarkers have to be corroborated by standard clinical trials in independent PDAC patients as well as in high-risk controls [37]. A multicenter study on analysis of 230 human tissue samples and 1011 plasma samples has suggested Keratin 5 (KRT5) and Versican (VCAN) as potential biochemical markers for early diagnosis and clinical surveillance of PDAC [38].

## 6. PDAC Preventive Measures

Although there are treatments (surgery, chemotherapy, immunotherapy and radiotherapy) for cancers [17,39], the supportive dietary and microbial therapies may offer additional survival advantages and protection against PC [40,41]. The first-line treatment, a combination chemotherapy called NALIRIFOX approved by USFDA, has been recommended for metastatic PC patients who have not received any previous treatment.

Given that PC might be the second leading cause of cancer-related morbidities and mortalities by 2030, there is an urgent need for alternative therapies, especially dietary interventions that are convenient, accessible and tolerant to patients. The aim of dietary and microbial interventions is to enhance the susceptibility of pancreatic tumor cells to chemotherapy, such as gemcitabine.

### 6.1. Dietary Interventions

Vegetarian foods promote gut microbial diversity and functional competence. The dietary plant ingredients, viz., carbohydrates, lipids, proteins, minerals, vitamins, and bioactive phytochemicals (anthocyanins, polyphenols, terpenoids, isoflavanoids, flavonoids, carotenoids, phytoestrogens, phytosterols, essential oils, omega 3 fatty acids, anthocyanins, and resveratrol, are crucial to nutrition, immunomodulation and overall well-being through multiple modes of action (Table 1)). Being naturally available in wide varieties of pulses, vegetables, nuts, fruits and grains, medicinal, plant-origin beverages and decoctions, the appropriately selected phytonutrients may have a crucial role in cancer deterrence, and avert the adverse effects associated with chemotherapy and radiotherapy. Further, compared to commercial nutraceuticals, the dietary phytonutrients are fairly inexpensive, hence also reducing healthcare costs. The beneficial or pharmacological effects of phytonutrients include anti-inflammatory, protection from UVB-induced carcinogenesis, anti-oxidative, anti-aging, anticancer, hepatoprotective, hypolipidemic, anti-hypertensive and immunomodulation [42]. Gut microbial metabolites of phytonutrients have enhanced bioavailability and bioactivities more than their precursors, hence microbial metabolism of ingested phytonutrients paves the ways to enhance therapeutic eminence of the dietary ingredients [42,43,44,45].

### 6.2. Vegan Diets and Phytotherapeutics

The bioactive metabolites in spices, medicinal plants and herbal beverages have anti-inflammatory, anti-oxidative, antimicrobial and anti-proliferative activities. The turmeric (*Curcuma longa*), also known as “Indian Saffron”, rhizome is used as spice in cooking and as traditional ethnomedicine for centuries. Curcumin, the bioactive components of turmeric, have powerful anti-inflammatory, anti-oxidative, anticancer and overall health benefits [44,46,59].

A recent bioinformatics, transcriptomic and molecular docking analysis and dynamics of effects of curcumin on human PC cell line (i.e., PANC-1 cells) have shown that 40 µg/mL of curcumin significantly inhibited the PANC-1 growth. The curcumin not only regulates cell cycle progression in PANC-1 cells, it down-regulates the expression of Cyclin D1, AKT serine/threonine kinase (AKT1), HRas proto-oncogene (HRAS), and epidermal growth receptor (EGFR), in addition to impeding the energy metabolism reprogramming and associated downstream signaling pathways that affect tumor cell metastasis and spreading [60]. Studies using cell lines (BxPC3, MiaPaCa2 and Panc1 PDACs) and xenograft mouse models have shown that curcumin sensitized the chemoresistant, i.e., gemcitabine-resistant, PDAC cell lines by inhibiting the Polycomb Repressive Complex 2 (PRC2) subunit (the Enhancer of Zeste Homolog-2, EZH2) and long non-coding RNAs (lncRNA) PVT1 expression, and prevented the spheroids development and several self-renewal driving genes. The curcumin suppressed the growth of gemcitabine-resistant tumors in mice models. The study underscores the prospective use of curcumin and standard drugs to overcome the chemoresistance in PDAC [48].

Biotransformation of dietary phytonutrients including ellagitannins, isoflavones, and lignans during their passage through the GI tract is critical to generate bioactive metabolites [61]. The sulphur-enriched cruciferous vegetables, such as cabbage, cauliflower, broccoli, and watercress which contain a phytochemical, the glucosinolate (GLS), are of interest. The myrosinase-producing intestinal bacteria transform the GLS to isothiocyanates such as sulforaphane with powerful biological and chemo-preventive properties [30,42,61].

Genistein is a bioactive aglycone isoflavonoid found in soy (*Glycine max*) and soy by-products (soy milk, tofu, soy flour, miso, and natto), and some other legumes such as pulses, sprouted grams, red clover, alfalfa, currants and resins [61]. The genistein exhibits a broad range of biological activities including anticancer effects by halting cell growth, promoting cellular apoptosis, blocking angiogenesis and dispersal of metastasis through anti-inflammatory signaling, induction of cell arrest at G2/M phase, inhibition of angiogenesis in tumor, apoptosis induction through modulating Bax/Bcl-2 and caspases activation, inhibition of intracellular signaling pathway PI3K/Akt, NF-kB and tyrosine kinase [49,61,62].

Because genistin, the glycoside form of isoflavonoid has low permeability, it is hydrolysed to genistein by intestinal bacteria to enhance its absorption and bioactivity [42]. *Asaccharobacter celatus*, *Adlercreutzia equolifaciens*, *Slackia isoflavoniconvertens*, *Slackia equolifaciens*, and *Enterorhabdus mucosicola* dwelling in the human intestine metabolize isoflavones (daidzein and genistein) to generate another biological active potent isoflavone, the equol, which has potential pro-health effects [61,63].

Anticancer efficacy of genistein has been examined using PANC-1 cell line and an orthotopic mice model. A nanosize phytotherapeutic delivery system, named HA/Gen-FO-Cub, consisting of genistein, frankincense essential oil (FO), within cubosomes coated with bioactive ligand hyaluronic acid (HA), was developed to boost anticancer effects of genistein. HA/Gen-FO-Cub improved cellular uptake and anti-migratory effects, improved anti-tumor effects in the orthotopic cancer model, suppressed tumor growth and down-regulated NF-kB and VGEF (2.9- and 1.8-fold, respectively) [49]. A combination of phytoestrogen Biochanin A and atorvastatin attenuated the anticancer effects, induced apoptosis and down-regulated cell cycle-associated proteins and invasiveness of PANC-1 cells [51].

Thymoquinone (TQ) in plants such as (*Nigella sativa* L., *Monarda fistulosa* L., *Satureja montana* L., and *Thymus vulgaris*) has medicinal properties. The cellular and molecular biological targets demonstrate the rationale of using TQ as an anti-neoplastic agent to prevent PDAC, hence enhancing the patient survival after surgical resection.

The TQ (25–100 µM, 24 h), along with α-hederin, exhibited a variable toxicity and anti-proliferative effects on different cancer cell lines, viz. human cancer cell lines: pancreas carcinoma (MIA PaCa-2), lung carcinoma (A54), larynx epidermal carcinoma (Hep-2). Hep-2 cells were most sensitive to treatments as indicated by high rates of apoptosis in these cells [52]. Similarly, TQ (10 µM) enhanced sensitization of PC cells to gemcitabine, reduced cell growth, increased apoptosis, increased NF-kB, and down-regulated Bcl-2, Bcl-xL, COX-2, survivin, and XIAP and the associated prostaglandin E2 (PGE2) [53]. TQ (10–50 µM) inhibited cell viability and proliferation, induced partial G2 cell arrest in a dose-dependent manner, up-regulated p53, and down-regulated Bcl-2, reduced HDACs activity, diminishing the HDACs 1, 2, and 3 by 40–60% [54].

PANC-1 cancer cells treated with TQ (5 µM to 35 µM) and gemcitabine (0.01 µM to 50.0 µM) (24 h) led to inhibition of cell migration, invasion and metastasis, increase in cell apoptosis, and improved sensitivity to gemcitabine action through regulation of ECM production through the TGBβ/Smad pathway wherein HIF-1α plays a key role. The TQ and gemcitabine were found to be more effective in preventing PANC-1 cells’ growth and migration [56].

Metagenomic and metabolomic profiling have shown that tryptophan microbial metabolite, the indole-3-acetic acid (3-IAA), was high in PDAC patients who have responded to cancer treatment. FMT, short-term dietary manipulation and orally administered 3-IAA improved chemotherapy adeptness through neutrophil-derived myeloperoxidase activities in humanized gnotobiotic mice models of human PDAC [58]. In human cohorts as well, a correlation was observed between 3-IAA levels and anticancer outcomes of PDAC, thus implying the importance of nutritional interventions to treat and improve the health of cancer patients [58].

Although further studies are suggested to explore the molecular mechanisms of anticancer effects, preliminary studies have shown that a nutraceutical, the Celergen, from marine resources reduced IL-6 expression and the IL-6 receptors in PC cell line (PSN-1) [11]. A study on 76 patients with metabolic syndrome has shown that a sturgeon-derived bioactive compound, named LD-1227, beneficially modulated nuclear receptors controlling metabolic functions in patients with metabolic syndrome, and hence could be integrated into a category of preventive medicines and supportive therapies [64].

### 6.3. Microbial Therapies

The microbial therapies or probiotics restore and enrich the gut microbial diversity, which is critical not only in digestion, gastrointestinal (GI) biotransformation and detoxification of certain anti-nutritional dietary components, but also synthesize nutrients such as vitamins, antimicrobial peptides, bacteriocins, neurotransmitters, modulate immunity and confer multiple health benefits [4,43,65].

The probiotics enrich gut microbial balance, suppress dysbiosis, maintain intestinal homeostasis, hence probiotics, FMT and fermented foods may have positive effects in PC patients and improve their quality of life. Although outcomes are variable and need further insights, the GI microbial modulation by FMT, probiotics, prebiotics, dietary manipulations, high-fiber diets, nutraceuticals and antibiotics has the potential to improve PDAC therapies’ outcomes [66].

Appropriate designer probiotics developed through genetic engineering or genome-editing or multi-omics approaches may be used to deliver drug load to anaerobic milieu of solid tumors to which the conventional therapies are less efficient [67]. Microbiome engineering, designer gut microbiota as futuristic probiotics, and microbial genome-editing hold promise to mitigate cancer resistance to existing therapies [20,27].

Importantly, we foresee the emerging field of co-biotics, postulated few years ago [68], the dual modulators of gut microbiota, as well as the host, will have a restorative and regulatory role in multiple health problems including cancers. However, most studies have been conducted in vitro models using cancer cell lines and in vivo by using animal models.

### 6.4. Fecal Microbiota Transplantation

Given that microbiome affects the efficacy of chemotherapy and immunotherapy, the future research should also focus on utility of microbiome as a screening tool to establish personalized medicine approaches [69]. FMT has been shown to increase chemotherapy efficacy in mice models. Compared to control and other treatments, the animals subjected to FMT and 5-flurouracil (5FU) treatment had a reduction in overall tumor volume, reduced pro-inflammatory cytokines (TNF-α, and IL-6), increased anti-inflammatory cytokine (IL-10), significantly high levels of blood plasma and fecal short chain fatty acids (SCFAs), all effects leading to an increase in survival rates (50%) of animals [70]. The anticancer effects were attributed to FMT-induced modulation of intestinal microbiota and microbial metabolism [70].

Up-regulation of gut symbionts such as lactic acid bacteria (LAB) may be a strategy to promote GI health and avert the adverse effects of GI diseases [40]. FMT from enrichment environment (EE) significantly inhibited tumor growth in subcutaneous and orthotopic PDAC mice models housed under standard environment. The anticancer effects of FMT are attributed to regulation of intestinal microbiota, reducing cytotoxic and pro-inflammatory metabolites, improving dysregulation of intestinal microbiota, promoting gut microbial diversity, especially enrichment of LAB such as *Lactobacillus reuteri*. Treatment with *L. reuteri* significantly suppressed the PDAC tumor growth and promoted natural killer cell infiltration into the tumor environment [40]. Although FMT has beneficial effects on a number of health problems, reports are scarce on its use to prevent PDAC.

Heterogeneous classes of plant fiber and their GI metabolites such as SCFAs modulate gut microbial ecology and impact host health. The strengthened or enriched gut microbiota is crucial to promote anti-PC immunotherapy and regulate immune checkpoints such as PD-L1 and CTLA-4. Butyrate promotes PC cell differentiation and inhibits their invasion and metastasis [71]. Epidemiological evidence indicates that butyrate reduced occurrence of various types of cancer. Notably, the population and diversity of butyrogenic microbes (e.g., *Ruminococcus* and *Faecalibacterium*) is diminished in PC patients, and had distinct microorganisms and microbial metabolic signatures [72].

### 6.5. Life Style Measures

Life style has a prominent effect on overall health and well-being of an individual. A case–control study on analysis of exposome and its interaction with genetic susceptibility to PDAC risk has shown that alcoholism, tobacco, cigarette smoking, chronic psychological stress, sedentary behavior [39], age, ethnicity, gender, environmental exposure, genetic predispositions, and life style diseases such as diabetes (T1D, T2DM, T3DM), hypercholesterolemia, and chronic pancreatitis adversely affect health and life [10]. Ironically, the prevalence of these risk factors is increasing on a global scale.

Refined sugar and sugar-sweetened beverages are linked to increased risk of PC, while non-alcoholic pancreatic diseases and central adiposity contribute through chronic inflammation and insulin resistance. Moreover, it has been demonstrated that patients with chronic pancreatitis have a shortage of dietary antioxidants and have impaired monocyte oxidative burst capacity which together serve as subtle risk factors of the cancer [73]. The gut microbiome and dysbiosis affects PC through the gut–pancreas axis by causing inflammation and carcinogenesis [74]. Healthy and active life styles and vegetarian diets may have positive effects in patients suffering from pancreatitis and pancreas-related problems.

## 7. Outlook and Challenges

The rise in cancer incidences is a matter of serious concern. The efforts to decrease cancer are impeded by inadvertent or inevitable exposure to dietary, respiratory and environmental hazards. Investments in terms of research and development grants are required to expand the infrastructure to understand cancer biology, identify robust biochemical and molecular biological markers in addition to already existing markers (Table 2). This will help in developing cost-effective targeted therapies, thus restraining the escalating global cancer burden. Further, the studies on PC at the molecular biological levels are still far from being sufficient, implying the need for comprehensive studies based on laboratory models, clinical trials and meta studies.

However, it is a challenging task, and proofs are still lacking on the role of natural microbiome and its metabolites on the onset and prevention of PDAC. Conventional, engineered and synthetic probiotics, and the bioactive phytonutrients consumed through pharmaceuticals, functional foods, and herbal beverages might be the prospective interventions to prevent diseases [75]. The microbial modulation has its own limitations due to variable composition and depends on the individual’s unique microbiota, which affect the treatment outcomes.

Antibiotics, surgery including robotic surgery, laparoscopic pancreatoduodenectomy and neo-adjuvant chemoradiotherapy have reduced the mortality associated with pancreatic surgery and managed the PDAC [17,19,70]; the high-risk PDAC patients will benefit from diagnosis of pre-malignant intraepithelial neoplasia, intraductal papillary mucinous neoplasms and mucinous cystic neoplasms. The futuristic efforts should focus on understanding how safely and effectively we can manipulate the existing microbiome, and enhance the effectiveness and bioavailability of phytochemicals taken through foods, beverages and pharmaceuticals. The clinical management of PDAC depends on advancement of the diseases, the stage at which it is diagnosed and overall health status of the patient. Future studies need to emphasize optimal type, dose and timing of intervention for microbial therapies and dietary supplements to explore precise interventions to prevent PDAC.

**Table 2:** Summary of prospective biomarkers in diagnosis and monitoring of PDAC.

Marker	Salient Features, Limitations and Recommendations (References)
CA 19-9	A mucin-like glycoprotein discovered on the cancer cell surface, duly approved by USFDA to diagnose PDAC. The CA 19-9 has 79–81% sensitivity, and 89–90% specificity, and yields better results when combined with other markers [76]. Clinical utility is limited by non-specificity in certain cases, leading to false-positives in benign biliary conditions, false-negatives in cells negative for Lewis enzymes. The sensitivity is low for early-stage detection [77].
CEA	CEA is a protein-based tumor marker, which can be used alongside CA 19-9 for PDAC prognosis [78]. The sensitivity ranges from 44.2 to 54.0%, which is higher than CA 19-9. Compared to CA 19-9, the CEA is a more robust marker of PDAC [79]. As non-cancerous conditions (bowel diseases, infections and cancers) may affect the CEA expression levels, it is not a reliable universal PDAC diagnostic marker.
sAXL	A protein fragment used as novel plasma biomarker to diagnose PDAC, HCC, CP and glioblastoma. sAXL is used to differentiate PDAC from chronic pancreatitis, accuracy is higher than CA 19-9 [80].
MIC-1	Also known as GDF15, belongs to TGF-superfamily. Serum MIC-1 acts as biomarker of prostate cancer, PC, CRC, metastasis and some inflammatory conditions, and tumor-promoting inflammation. In combination with CA 19-9, the MIC-1 is used for early-stage detection of PDAC [81]. Compared to CA 19-9 sensitivity (59%), the serum MIC-1 has higher sensitivity (71%) [81].
ctDNA, exosomes:	*KRAS* mutations improve detection of stage 1 and 2 PDAC. Methylation profiling of ctDNA is used to develop an assay (named PDACatch) which serves as a sensitive and non-invasive tool for PDAC detection [82].
miRNA Panels	Better detection of PDAC during early stages using miRNAs (e.g., miR-143/miR-30e) based on cellular metabolites, e.g., proline, creatinine, palmitic acid, etc. The miRNA, being stable in blood circulation, serves as a non-invasive marker for early detection of tumor-specific profiles [83,84].
Microbial signatures	The microbial profiles serve non-invasive biomarkers of tumors, combination of gut microbiome profiling and CA 19-9 found to improve PDAC detection [76].

## 8. Conclusions

The PDAC is an inflammation-driven cancer triggered by multiple factors. The precise mechanisms underlying the PDAC pathogenic are yet to be unraveled. Preclinical and clinical evidence suggest that the gut microbiome influences cancer-associated inflammation and serves as a mediator of PDAC development. The elucidation of interplay between inflammation, diet and microbiome dynamics in the context of pancreatic carcinogenesis will pave the ways to develop strategies to prevent and treat PDAC. A thorough and better understanding of the risk factors and symptoms associated with the PDAC is essential for both health professionals and the general population, especially the persons susceptible to health hazards that weaken the immunity. This will help to diagnose the disease at early stages and follow appropriate preventive measures besides standard therapies available at the moment.

## Figures and Tables

**Table 1:** Summary of dietary phytochemicals, nutraceuticals and botanicals and their anti-PDAC properties. The microbial metabolites of selected phytonutrients are more bioactive than their precursors. Not all the phytochemicals and their metabolites are useful. Amygdalin and mimosine (a non-protein amino acid), for instance, generate toxic gut microbial metabolites. The intake of these phytochemicals is low in humans, hence adverse effects are squat.

Sl. No.	Bioactive Phytochemicals (Source)	Modes of Study	Mechanisms, Inferences and Recommendations (References)
1.	Curcumin (*Curcuma longa*)	Bioinformatics, in silico analysis, transcriptome sequencing, and molecular docking of interaction of curcumin and PANC-1 cell line	Significant inhibition of growth and proliferation PANC-1 by 40 µg/mL curcumin, down-regulation of Cyclin D1 (CCND1), AKT serine/threonine kinase (AKT1), HRas proto-oncogene (HRAS), and epidermal growth receptor (EGFR) [46].
		In vitro, using BxPC-3 cell line, and HPDE-6 cell line (as control)	Activation of ATM/CHK1 pathway, significant arrest of BxPC-3 cells in G2/M phase, and induction of apoptosis through a single exposure of 2.5 µM curcumin for 24 h, the HPDE-6 cells remain unaffected [47].
		In vitro (gemcitabine-resistant PDAC cell lines) (BxPC3, MiaPaCa2 and PANC-1 PDACs) In vivo (mice models)	Curcumin-mediated sensitization of PDAC cells to chemotherapy, prevention of spheroids development and several self-renewal driving genes, suppression of gemcitabine-resistant tumor growth [48].
2.	Genistein (Soy and soy products, some pulses and legumes)	In vitro (PANC-1), in vivo (orthotopic cancer model)	Development of novel nanosize FO bioactive cubosome (HA/Gen-FO-Cub) to deliver and actively target the cancer cells, and anti-tumor effects (in vivo). Documentation of 2.5-fold drop in tumor growth, and down-regulation of NF-kB and VEGF [49].
		In vitro, MiaPaCa2 and PANC-1 PDACs cells	Induction of morphological alterations at cell cycle arrest in G0/G1 phase in cancer cells, apoptosis in dose-dependent manner, ROS-mediated apoptosis in cancer cell mitochondria, and regulation of STAT3 signaling pathway [50].
3.	Biochanin A (some pulses and legumes)	In vitro (PANC-1 cells)	Biochanin A and atorvastatin-mediated anticancer effects, apoptosis and down-regulated cell cycle-associated proteins and invasiveness of PANC-1 cells [51].
4.	Thymoquinone (TQ) (*Nigella sativa* L., *Monarda fistulosa* L., *Satureja montana* L., *Thymus vulgaris*)	Meta-analysis of published data In vitro (human cancer cell lines: pancreas carcinoma (MIA PaCa-2), lung carcinoma ((A54), larynx epidermal carcinoma (Hep-2)))	Dose (25–100 µM, 24 h) and time-dependent inhibitory effects of TQ and α-hederin on cancer cell lines, enhanced cytotoxicity and reduced cell proliferation. Hep-2 cells were most sensitive to treatments [52].
		In vitro, cancer cell lines (MIA PaCa-2, AsPC-1, BxPC-3, HPAC)	Sensitization of pancreatic cells to gemcitabine, reduction in cell growth, increased apoptosis, increase in NF-kB, and down-regulation of Bcl-2, Bcl-xL, COX-2, PGE2, survivin, and XIAP [53].
		In vitro, human PDAC cell lines (AsPC-1, MIAPaCa-2)	TQ (10–50 µM)-mediated inhibition of cell viability and proliferation, partial G2 cell arrest in dose-dependent manner, up-regulation of p53, and down-regulation of Bcl-2, reduction in HDACs activity diminishing the HDACs 1, 2, and 3 by 40–60% [54].
		In vitro, HS766T PADC cells	Time- and dose (25–75 µM, 24 h)-dependent inhibition of MCP-1, TNF-α, IL-1β and Cox2 at 24 h of treatment [55].
		In vitro, PANC1 cells	Inhibition of cell migration, invasion and metastasis, increase in cancer cell apoptosis, and improved sensitivity to gemcitabine in combination with TQ [56].
5.	Glucosinolates (GLSs)	In vivo (rats)	*L. rhamosus* GG-mediated complete transformation of *Tropaeolum tuberosum* glucosinalbin and glucotropaeolin, 46.7% of glucoaubrietin, and their absorption and metabolism. The probiotic-mediated biotransformation is beneficial to consumers [57].
6.	Celergen	In vitro (cell lines, PSN-1)	Reduction in IL-6 and IL-6 receptors in PSN-1 cells [11].
7.	Tryptophan and 3-IAA (GI microbial metabolite)	Shotgun metagenomic sequencing and metabolomics analysis	Improvement in chemotherapy outcomes through FMT, short-term dietary manipulation of tryptophan and oral administration IAA in humanized mouse models of PDAC, observation of correlation between IAA and treatment of PDAC human patients [58].

Abbreviations: 3-IAA—indole-3-acetic acid; FO—frankincense oil; Gen—genistein; HA—hyaluronic acid; HPDE—human pancreatic ductal epithelial cells; IAA—indole acetic acid.

## Data Availability

No new data were created or analyzed in this study.
